# The Effectiveness of a Brief Telehealth and Smartphone Intervention for College Students Receiving Traditional Therapy: Longitudinal Study Using Ecological Momentary Assessment Data

**DOI:** 10.2196/33750

**Published:** 2022-06-29

**Authors:** Madison E Taylor, Olivia Lozy, Kaileigh Conti, Annmarie Wacha-Montes, Kate H Bentley, Evan M Kleiman

**Affiliations:** 1 Department of Psychology Rutgers, The State University of New Jersey Piscataway, NJ United States; 2 Clinical Psychology Northwell Health New Hyde Park, NY United States; 3 Department of Psychiatry Massachusetts General Hospital Harvard Medical School Boston, MA United States

**Keywords:** college students, digital mental health, brief interventions

## Abstract

**Background:**

Brief interventions such as mental health apps and single-session interventions are increasingly popular, efficacious, and accessible delivery formats that may be beneficial for college students whose mental health needs may not be adequately met by college counseling centers. However, no studies so far have examined the effectiveness of these modes of treatment for college students who are already receiving traditional therapy, despite it being common among this population.

**Objective:**

The aim of this study was to compare the differences in self-reported momentary negative affect between college students in therapy and not in therapy who received a brief single-session intervention delivered by counseling center staff and a supplemental mobile app.

**Methods:**

Data for this study were drawn from E-Manage, a brief mobile health intervention geared toward college students. Participants in the study were 173 college students who indicated whether they had received therapy. We conducted a multilevel model to determine whether there were differences between those in therapy versus not in therapy in negative affect reported throughout the study. Following this, we conducted multilevel models with therapy status as the predictor and negative affect as the outcome.

**Results:**

Results of the multilevel model testing showed that the cross-level interaction between the time point (ie, pre- vs postexercise) and therapy status was significant (*P*=.008), with the reduction in negative affect from pre- to postexercise greater for those in therapy (*b*=–0.65, 95% CI –0.91 to –0.40; *P*<.001) than it was for those not in therapy (*b*=–0.31, 95% CI –0.43 to –0.19; *P*<.001). Therapy status was unassociated with both the pre-exercise (*b*=–1.69, 95% CI –3.51 to 0.13; *P*=.07) and postexercise (*b*=–1.37, 95% CI –3.17 to 0.43; *P*=.14) ratings of negative affect.

**Conclusions:**

These findings suggest that app-based and single-session interventions are also appropriate to use among college students who are receiving traditional therapy. A randomized controlled trial comparing students receiving therapy to students receiving therapy and E-Manage will be necessary to determine to what extent E-Manage contributed to the reductions in negative affect that therapy-attending college students experienced.

## Introduction

It has been well established that traditional models of mental health care are inaccessible to many of those who need it [1]. Various factors contribute to this inaccessibility, including an insufficient number of providers to meet demands [2], lack of affordability [3], and stigma [4]. Furthermore, traditional models of mental health care do little to decrease strain on overburdened mental health care systems [5]. Thus, there is a distinct need for innovative and scalable interventions that can increase access to mental health care and reduce burden on service providers.

Brief interventions using mobile technology (ie, apps) and interventions designed to be completed in one sitting (ie, single-session interventions) are becoming popular and efficacious delivery formats for mental health needs that help make mental health care more accessible [6-9]. These accessible modes of treatment may be particularly beneficial for college students, who experience high levels of mental disorders [10,11]. Worldwide, about 1 in 4 college students meet the diagnostic criteria for a mental disorder within a given year [12], with the rates for some disorders having doubled over the past decade [13]. Despite these upward trends, college counseling centers have struggled to keep up with the growing demands for individual therapy due to institutional constraints, such as using insufficient amounts of staff members to meet student needs [11,14]. In light of the budgetary constraints on college counseling centers, efficient delivery formats such as mental health apps and single-session interventions are appealing to address increased student demand, especially due to growing evidence of their effectiveness for and ease of dissemination to college students [15-20].

However, no studies so far have examined the effectiveness of these brief mobile interventions for college students who are already receiving mental health treatment. Approximately 9% of college students use counseling center services within a given year [10], and 15% of students have received therapy at some point in their lives [21]. Understanding the effectiveness of mental health apps and single-session interventions in college students who have received therapy is important for determining how these tools may be disseminated among college students. There are two possibilities that could arise. If mental health apps and single-session interventions are found to be just as effective or more effective for students who are currently receiving (or have previously received) therapy (compared to those never in therapy), these treatment modalities could be offered as a beneficial adjunct to their care. Mental health apps may add some benefit to those who have had therapy because they have prior experience both in terms of socialization (ie, familiarity with what therapy is like) and familiarity with specific content. If these modes of treatment modalities are not as effective for students currently or previously in therapy, counseling centers may want to direct their dissemination to students who have not sought out traditional treatment.

The purpose of this study is to examine if there were any differences in the effectiveness of a brief single-session intervention delivered by counseling center staff and mobile app between college students in therapy and those not in therapy. To accomplish this, we analyzed data collected from 173 participants in E-Manage [22], a brief 90-minute workshop based on the Unified Protocol [23] with a supplemental mobile app implemented in a northeastern public university’s counseling center. We compared the differences in self-reported momentary negative affect across 8 weeks post workshop between students reporting attending therapy and students reporting not attending therapy. We also examined whether or not these differences were due to differences in baseline negative affect. We hypothesized that there would be differences in the change in negative affect between the two groups. More specifically, we hypothesized that students attending therapy would have greater reductions in negative affect from E-Manage than those not in therapy due to prior socialization to the skills introduced in E-Manage through their therapy treatment.

## Methods

### Participants

Data for this study were drawn from a Registered Clinical Trial (NCT04636151) of E-Manage, a brief mobile health intervention geared toward college students [18]. Participants in the study were 173 college students, of the original study sample of 177, since 4 participants did not indicate whether they had received therapy previously and were thus excluded from these analyses. Regarding gender, 78.6% (n=136) of the sample identified as cisgender female, 16.8% (n=29) as cisgender male, and the remainder identified as nonbinary or gender nonconforming. Regarding race, the sample was 42.8% (n=74) White, 34.1% (n=59) Asian, 10.4% (n=18) Black/African American, 8.7% (n=15) more than one race, and the remainder chose not to disclose race. Regarding ethnicity, 13.3% (n=23) identified as Hispanic or Latinx. Regarding prior therapy exposure, 31.8% (n=55) of the sample reported previously attending therapy.

### Ethics Approval

All study materials and procedures were approved by Rutgers, The State University of New Jersey’s Institutional Review Board (Federal Wide Assurance Identifier FWA00003913).

### Analytic Strategy

We first created a negative affect composite variable using the four negative affect variables asked at both pre- and postexercise (agitated, angry, hopeless, burdensome). We then conducted two sets of multilevel models in the *lme4* R package. The first set of models was conducted to determine whether there were differences between those in therapy versus not in therapy in negative affect reported throughout the study. We conducted multilevel models with therapy status as the predictor and negative affect as the outcome. We conducted separate models for the pre-exercise ratings and the postexercise ratings, given that we were hypothesizing differences in pre-post ratings of negative affect between groups and did not want to introduce this expected difference as a confound to these analyses. The second analysis tested our primary hypothesis. We conducted another multilevel model that included a time point (ie, pre- vs postexercise rating) at the observation level, therapy status at the person level, and the cross-level interaction between the two. We probed the simple slopes of the significant interaction using the *reghelper* package.

## Results

Results of the multilevel models exploring whether those in therapy reported more negative affect than those not in therapy suggested that this was not the case. Therapy status was unassociated with both the pre-exercise (*b*=–1.69, 95% CI –3.51 to 0.13; *P*=.07) and postexercise (*b* =–1.37, 95% CI –3.17 to 0.43; *P*=.14) ratings of negative affect.

**Figure 1:**
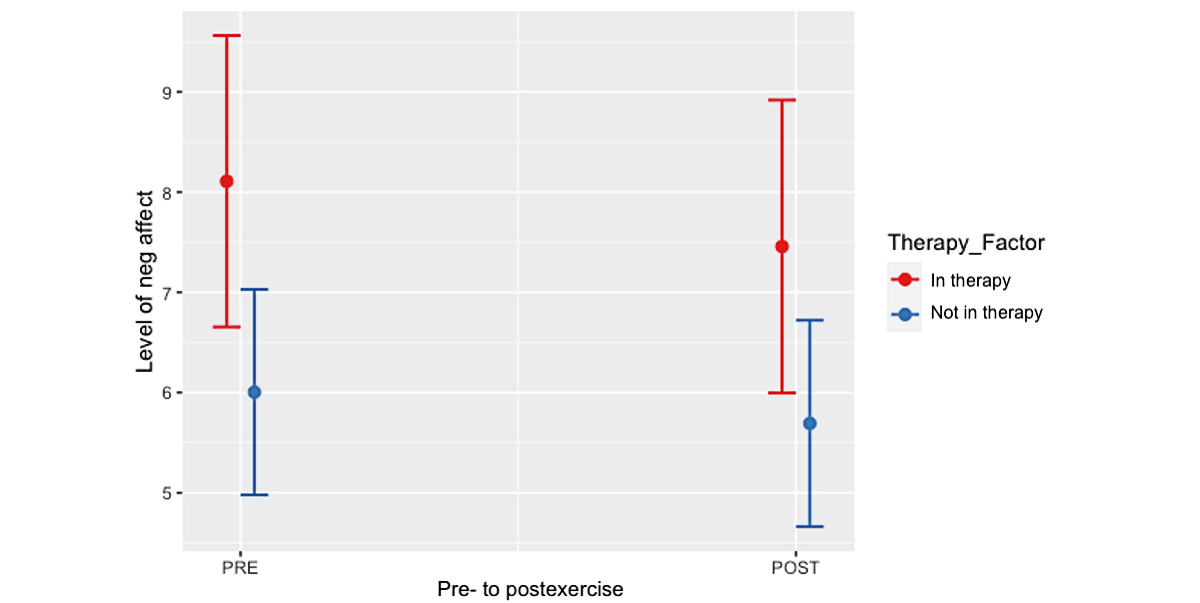
Changes in negative affect from pre- to postexercise for college students in therapy versus not in therapy. neg: negative.

Results of the multilevel model testing our primary hypothesis showed (Table 1) that the cross-level interaction between the time point (ie, pre- vs postexercise) and therapy status was significant. When we further plotted (Figure 1) and probed the model, we found that the reduction in negative affect from pre- to postexercise was greater for those in therapy (*b*=–0.65, 95% CI –0.91 to –0.40; *P*<.001) than it was for those not in therapy (*b*=–0.31, 95% CI –0.43 to –0.19; *P*<.001).

**Table 1:** Results of the multilevel model predicting negative affect.

Predictors		Estimates (95% CI)	*P* value
(Intercept)		8.11 (6.65 to 9.56)	<.001
Pre vs post (ref=pre)		–0.65 (–0.86 to –0.44)	<.001
In therapy (ref=in therapy)		–2.10 (–3.88 to –0.33)	.02
Pre/post X therapy		0.34 (0.09 to 0.59)	.008
**Random effects**			
	Within-person residual variance (*σ*^2^)	16.64	N/A^a^
	Between-person residual variance (*τ*_00_)	29.66	N/A
	Intraclass correlation coefficient	0.64	N/A
	N_ID_	166	N/A
	Observations, n	22,846	N/A
	Marginal *R*^2^ (conditional *R*^2^)	0.020 (0.648)	N/A

^a^N/A: not applicable.

## Discussion

### Principal Findings

Mental health apps and single-session interventions show promise as solutions to the growing mental health demands of college students. However, little has been done prior to this study to examine the effectiveness of these modes of treatment for the sizeable amount of college students who are already receiving therapy. The aim of this study was to compare the effectiveness of a brief workshop with a supplemental mobile app between college students who were concurrently attending therapy and students who were not. We found that both college students who had prior exposure to therapy and those who did not reported significant reductions in negative affect over the 8-week period. These reductions are in line with the results of prior studies examining digital mental health interventions in college students, which have found such interventions to be effective in reducing depression, anxiety, and stress for students seeking services at college counseling centers [17,18]. Those who did have exposure to therapy reported a greater reduction in negative affect in the 8 weeks following the workshop than students who were not currently in therapy. Additionally, there were no between-group differences in the level of negative affect across the study, suggesting that the greater reduction in negative affect that college students in therapy experienced cannot be explained by having a higher average level of negative affect.

One explanation for these findings is that students who have exposure to therapy are familiar with thinking about and addressing uncomfortable and sometimes distressing feelings while receiving treatment. Since some of the skills taught in the workshop may cause distress (eg, reflecting on negative thoughts while addressing cognitive distortions), college students with prior exposure to therapy may have been better equipped to handle this distress, allowing them to benefit more. Since we did not measure familiarity with therapy in this study, we are not able to examine if it is responsible for this difference. Future studies could assess familiarity with therapy to college students before they begin treatment to see if socialization to therapy is a mechanism of action. If it is a mechanism of action, future iterations of E-Manage and other mental health apps or single-session interventions could consider adding psychoeducation on what to expect in therapy at the beginning of the program to improve outcomes for students not attending therapy.

Additionally, college students with prior therapy exposure may have been previously exposed to the specific skills provided in this treatment. The skills taught in the E-Manage workshop and mobile app were based off the Unified Protocol, a form of therapy that combines elements of cognitive therapy, behavioral therapy, and mindfulness to help clients improve their ability to regulate distressing emotions [23]. Given the prevalence of cognitive behavioral therapy and mindfulness elements in therapy [24,25], it is highly possible that the prior therapy students received introduced them to the therapeutic content we provided. This familiarity could have led students with prior therapy exposure to be more receptive and engaged. We did not evaluate the content of the therapies students in this study were receiving. This makes us unable to determine if students in therapy learned skills similar to those they learned in the workshop/app, and thus if they were truly socialized to these skills prior to their participation in this study.

### Limitations

There are some additional limitations and future directions to consider when interpreting the findings of this study. First, due to lacking a therapy-only control group, we are unable to determine to what extent the reductions in negative affect that therapy-attending college students experienced were due to receiving E-Manage rather than changes resulting from receiving additional therapy. A randomized controlled trial comparing reductions in negative affect for students in therapy versus those in therapy with the workshop and app is still necessary to resolve this question. We are also unable to assess if E-Manage was able to lessen the time college students attending therapy needed to spend in treatment. This could also be addressed in a future randomized controlled trial comparing students attending therapy and receiving E-Manage with college students only attending therapy.

Additionally, several studies have noted ongoing concerns over the lack of fidelity monitoring and quality assurance when implementing mobile treatments into clinical settings, potentially hampering the scalability of these treatments post study [26,27]. While such measures were not the focus of this study, future work on E-Manage should focus on examining the treatment’s fidelity to the Unified Protocol when being delivered by college counselors to ensure the scalability of E-Manage. Finally, because we did not recruit from a clinical population, participants in the study likely had a range of symptom severity. Future studies will be needed to determine the effectiveness of E-Manage for different levels of symptom severity in college students.

### Conclusions

These findings suggest that brief single-session interventions and apps like E-Manage may be appropriate to use for college students receiving therapy as well as those not receiving therapy. This suggests that programs like E-Manage could be useful as a broader prevention framework in the college community. As more college students attend therapy prior to their freshman year [11], some students may have to terminate with their current therapist to relocate to their college, resulting in a gap in their mental health treatment. Due to its ease of dissemination, one practical application for brief technology-based treatments like E-Manage could be implementing them in programs for incoming freshmen, helping to bridge the gap for these students as they transition into college and locate new therapists. Another practical application for E-Manage, given the reductions in negative affect for students in therapy, could be to serve as a treatment adjunct in college counseling centers. It is possible that giving students in counseling centers access to programs like E-Manage could lead to more substantial gains during traditional treatment in a shorter period of time, improving counseling center’s ability to provide individual therapy services to other students. Integrating mental health apps and single-session interventions like E-Manage into routine care in counseling centers could ultimately lead to meaningful reductions in the burden placed on counseling centers.
